# The neural connectome of suicidality in adults with mood and anxiety disorders

**DOI:** 10.1038/s44220-024-00325-y

**Published:** 2024-10-04

**Authors:** Richard A. Bryant, Isabella A. Breukelaar, Thomas Williamson, Kim Felmingham, Leanne M. Williams, Mayuresh S. Korgaonkar

**Affiliations:** 1 School of Psychology, University of New South Wales, Sydney, New South Wales Australia; 2 Brain Dynamics Centre, Westmead Institute for Medical Research, The University of Sydney, Westmead, New South Wales Australia; 3 Discipline of Psychological Science, University of Melbourne, Melbourne, Victoria Australia; 4 Department of Psychiatry and Behavioral Sciences, Stanford University, Stanford, CA USA; 5 Sierra-Pacific Mental Illness Research, Education and Clinical Center (MIRECC), VA Palo Alto Health Care System, Palo Alto, CA USA; 6 Discipline of Psychiatry, Sydney Medical School, Westmead, New South Wales Australia; 7 Department of Radiology, Westmead Hospital, Western Sydney Local Health District, Westmead, New South Wales Australia

**Keywords:** Psychology, Depression

## Abstract

Although suicide risk is a major public health issue, attempts to understand the neural basis of suicidality have been limited by small sample sizes and a focus on specific psychiatric disorders. This sample comprised 579 participants, of whom 428 had a psychiatric disorder (depression, anxiety or stress-related disorder) and 151 were non-psychiatric controls. All participants underwent structured clinical interviews, including an assessment of suicidality in the past month, and completed a functional magnetic resonance imaging scan. There were 238 (41.1%) participants who met criteria for suicidality and 341 (58.9%) were non-suicidal. Task-derived functional connectivity was calculated for 436 brain regions, comprising 8 intrinsic connectivity networks. Participants who were suicidal had decreased connectivity in a network of 143 connections across 86 brain regions. This pattern was characterized primarily by decreased connectivity within the visual, somatomotor and salience networks, between these networks, and also with the default mode and limbic networks. By adopting a transdiagnostic approach with a very large sample of individuals with mood disorders, anxiety and stress and non-psychiatric participants, this study highlights the hypoconnectivity that characterizes suicidality and points to altered connectivity within and between key networks involved in emotional, sensory and cognitive processes that are implicated in suicidal risk.

## Abstract (web summary)

In this study, the authors use a transdiagnostic approach to assess functional connectivity in individuals with and without a psychiatric diagnosis, showing hypoconnectivity in the default mode, visual and limbic systems associated with suicidality.

## Main

Suicide is a major cause of death worldwide^1^ and is responsible for approximately 800,000 deaths annually^2^. It is especially prevalent in younger people and in those with mental disorders. Preventing suicide is one of the highest global public health priorities^3^. Suicidal ideation and previous suicide attempts are key predictors of completed future suicide attempts^4–6^, with 60% of people with suicidal ideation transitioning to a suicide attempt within a year of the ideation onset^7^. Despite this, our understanding of the mechanisms underpinning suicidality is limited at this stage. Accordingly, there is an urgent need to understand the key factors that characterize suicidality.

In recent years there have been numerous attempts to characterize neural biomarkers of suicidality. Structural magnetic resonance imaging (MRI) analyses have reported that suicidality is associated with abnormalities in the dorsomedial prefrontal cortex, orbitofrontal cortex (OFC), amygdala, basal ganglia and insula^8–10^. Functional MRI (fMRI) paradigms of various tasks have indicated that individuals that attempt suicide can have abnormal activity in the lateral, medial and ventral prefrontal cortex, basal ganglia, limbic system and thalamus^11^. Only a few studies have reported altered task-based functional connectivity, particularly between fronto-striatal regions during reward tasks, fronto-limbic regions during emotion tasks, and between the dorsal anterior cingulate cortex and precuneus during conflict-processing tasks, which have been associated with suicide attempt and ideation symptoms^11–13^. In contrast, much attention has focused on intrinsic functional connectivity, which indexes the tonic state of the brain and potentially reflects intrinsic abnormalities in key neural networks. Intrinsic functional connectivity is typically measured when participants are at rest (that is, using task-free or resting-state fMRI) or alternatively can be derived from task-based fMRI after removing task-related variance from time-series data^14^. It is worth noting that almost all these studies have focused on suicidality in psychiatric disorders. In terms of depression, suicidality is associated with distinctive functional connectivity across a range of networks, including the default mode network (DMN)^15^, between the amygdala and insula, OFC, middle temporal gyrus, fronto-thalamic network, and insula–DMN. Numerous studies have examined suicidality in bipolar disorder, with the primary finding involving aberrant functional connectivity in the DMN^16^, fronto-thalamic connections^17^ and the salience network^18^.

Despite these advances, the understanding of resting-state connectivity in suicidality remains limited. Three key factors may contribute to the mixed findings in the literature. First, sample sizes are typically very small in published studies, with a substantial majority of studies including samples of less than 40 individuals who were suicidal^15–18^ (also see review in ref. ^11^). This limitation probably leads to insufficient statistical power to detect neural markers. Second, the vast majority of studies have focused on patients with either major depressive disorder (MDD) or bipolar disorder, thereby ignoring the fact that suicide attempts also occur in the context of schizophrenia, substance-use disorders, anxiety and personality disorders, and in people with no psychiatric disorders^11^. Finally, all but one^15^ of these previous studies only investigated suicidality-related differences in the DMN and salience and cognitive-control-related networks (frontoparietal and cingulo-opercular networks), without including other brain networks, which has created a bias in the field toward differences in these networks alone.

To overcome these limitations, this study examined the functional connectome of a very large cohort of individuals that comprised both healthy individuals and patients with a range of anxiety, mood and stress disorders. We used task-fMRI data collected from these cohorts to derive intrinsic functional connectivity across the entire connectome with the goal to identify distinctive whole-brain functional connectivity associated with suicidality, independent of clinical diagnoses. On the basis of previous work, we hypothesized that suicidality would be characterized by aberrant connectivity in the DMN, salience networks and fronto-thalamic network.

## Results

### Participant demographics

Of the 579 participants included in the analyses, 238 (41.1%) met criteria for suicidality and 341 (58.9%) were classified as non-suicidal. Of those showing suicidality, 168 thought they would be better off dead, 51 wished to harm themselves, 107 thought about suicide, 6 had a suicide plan, 2 had attempted suicide in the past month and 103 participants had a lifetime suicide attempt. However, there was a large overlap in responses across multiple measures, with 70% of participants responding to at least 2 items. The distribution of responses is shown in Supplementary Fig. 1. Table 1 summarizes the key characteristics of the sample. *t*-tests revealed significant differences in years of education and Depression, Anxiety and Stress Scale (DASS) scores between the suicidal and non-suicidal groups. Pearson’s chi-squared tests revealed significant differences in sex and clinical diagnosis between the two groups. Specifically, participants who were suicidal were more likely to be women, have slightly lower years of education, have a psychiatric disorder, and have higher levels of depression, anxiety and stress. Among the clinical diagnostic groups, all controls (standard, grief, trauma and mild traumatic brain injury (mTBI) controls) had the lowest percentage of suicidality, followed by those with social phobia. Prolonged grief and post-traumatic stress disorder (PTSD) had a prevalence of 50% suicidality, followed by a 60% prevalence in MDD and panic disorder. The highest prevalence of suicidality was found in those with complex PTSD and generalized anxiety at >80%.

**Table 1:** Participant characteristics

	Non-suicidal	Suicidal	*P* value	Total
	(*N* = 341)	(*N* = 238)		(*N* = 579)
**Age, yr**				
Mean (s.d.)	35.2 (12.4)	35.9 (12.6)	0.486	35.5 (12.5)
**Sex,** ***n*(%)**				
Female	161 (47.2)	148 (62.2)	<0.001	309 (53.4)
Male	180 (52.8)	90 (37.8)		270 (46.6)
**Education, yr**				
Mean (s.d.)	14.7 (2.84)	13.9 (2.98)	0.00172	14.4 (2.92)
**Suicidality,** ***n*** **(%)**				
Never	341 (100)	0 (0)	<0.001	341 (58.9)
Previous attempt (no current)	0 (0)	42 (17.6)		196 (33.9)
Current	0 (0)	196 (82.4)		42 (7.3)
**Diagnosis,** ***n*** **(%)**			<0.001	
Anxiety disorders	31 (9.1)	32 (13.4)		
GAD	2 (0.6)	9 (3.8)		11 (1.9)
Panic disorder	8 (2.3)	13 (5.5)		21 (3.6)
Social phobia	21 (6.2)	10 (4.2)		31 (5.4)
Depressive disorders	110 (32.3)	145 (60.9)		
MDD	99 (29.0)	135 (56.7)		234 (40.4)
Prolonged grief	11 (3.2)	10 (4.2)		21 (3.6)
Stress disorders	26 (7.6)	48 (20.2)		
PTSD	20 (5.9)	22 (9.2)		42 (7.3)
cPTSD	6 (1.8)	26 (10.9)		32 (5.5)
Controls	174 (51)	13(5.5)		
mTBI	31 (9.1)	5 (2.1)		36 (6.2)
Trauma controls	21 (6.2)	2 (0.8)		23 (4.0)
Grief controls	22 (6.5)	2 (0.8)		24 (4.1)
Healthy controls	100 (29.3)	4 (1.7)		104 (18.0)
**DASS anxiety**				
Mean (s.d.)	6.20 (7.30)	12.9 (7.95)	<0.001	9.01 (8.26)
**DASS depression**				
Mean (s.d.)	10.4 (10.5)	24.3 (10.2)	<0.001	16.2 (12.4)
**DASS stress**				
Mean (s.d.)	12.0 (10.1)	21.0 (8.88)	<0.001	15.8 (10.6)
**Fractional displacement**				
Mean (s.d.)	0.0834 (0.0347)	0.0847 (0.0343)	0.641	0.0839 (0.0345)
**Movement outliers, %**				
Mean (s.d.)	9.06 (8.97)	8.52 (8.77)	0.467	8.84 (8.89)

GAD, generalized anxiety disorder; cPTSD, complex PTSD.

### Connectome differences between participants who are suicidal and those who are non-suicidal

**Fig. 1:**
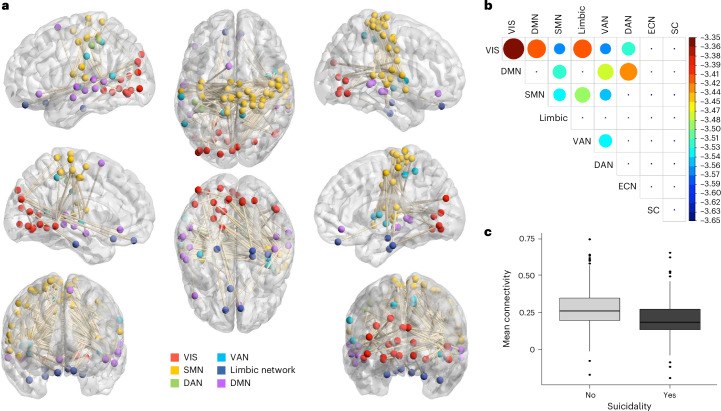
Network of connections showing decreased connectivity in participants who are suicidal. **a**, Identified network consisting of 86 nodes and 143 edges visualized on the brain surface. **b**, A heat map of the mean *t*-statistic of connections within and between the eight primary networks. Larger circles indicate a greater decrease in connectivity in the suicidal group. **c**, Mean difference in connectivity (Fisher’s z score) across the entire network identified by NBS. Boxplot data are presented as median value (centre line) plus 1st and 3rd quartile respectively (box bounds) with whiskers representing minimum and maximum values respectively defined by 1st or 3rd quartile plus 1.5 times the interquartile range and single points representing outliers. ECN, executive control network; SC, subcortex; VAN, ventral attention network. Panel **a** created with BrainNet Viewer.

**Fig. 2:**
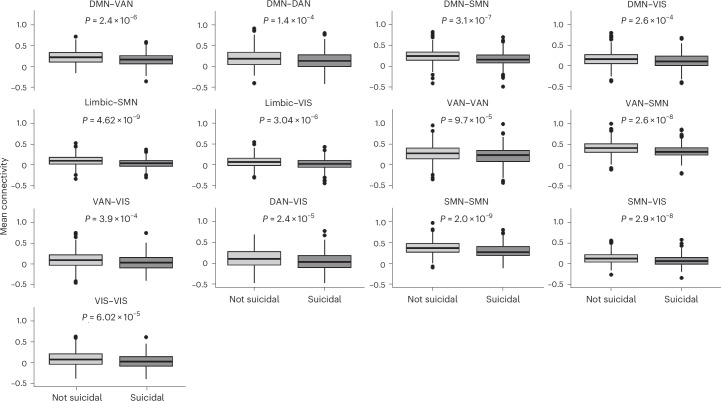
Mean functional connectivity estimates (Fisher’s z score) for each substantial intrinsic connectivity network (ICN) pair that distinguished Suicidal and Non-suicidal groups. Boxplot data are presented as median value (centre line) plus 1st and 3rd quartile (box bounds) with whiskers representing minimum and maximum values respectively defined by 1st or 3rd quartile plus 1.5 times the interquartile range and single points representing outliers. DMN, default mode network; DAN, dorsal attention network; SMN, somatomotor network; VAN, ventral attention network; VIS, visual network.

Using the network-based statistic (NBS), a network of 86 brain regions and 143 connections was identified. This network showed significantly decreased connectivity in participants who were suicidal compared with those without suicidality in the past month (*P* < 0.042, family-wise error corrected). This network was largely characterized by (1) decreased connectivity within the visual network (VIS), somatomotor network (SMN) and ventral attention/salience network (Fig. 1b, diagonal); (2) decreased internetwork connectivity between the VIS, SMN, DMN and ventral attention/salience network; (3) decreased connectivity between the VIS and SMN and the limbic network; and (4) decreased connectivity between the dorsal attention network (DAN) and the ventral attention/salience network, DMN and VIS (Figs. 1 and 2). Supplementary Table 2 and Fig. 2 summarize the direction of connectivity and percentage of edges within or between intrinsic connectivity networks (ICNs). A list of all the identified connections are provided in Supplementary Table 3.

### Differences in connectivity due to current suicidality

The effect of suicidality group (currently suicidal, previously suicidal and non-suicidal) was significant on functional connectivity for the identified connectome (*P* < 0.001), and post hoc (Tukey honest significant difference) tests showed that this was driven by a significant difference between both currently and previously suicidal groups compared with the non-suicidal group, but no difference was found between current and previously suicidal groups. Differences across each of the ICN pairs (Bonferroni-corrected *P* < 0.05) between the groups are reported in Supplementary Table 4. There was also no difference between currently suicidal participants with and without a suicide attempt, but both were significantly different from non-suicidal groups (*P* < 0.001; Supplementary Table 5).

### Differences in connectivity due to previous suicide attempts versus only ideation

There were no differences between participants who had attempted suicide and those who were non-attempers on total mean connectivity for the whole network (*F* = 1.0281, *P* = 0.3116) or in any ICN pairs at a corrected level (reported in Supplementary Table 6).

### Differences in connectivity due to clinical diagnosis

There was no significant interaction between diagnostic group and suicidality-related connectivity for the overall network (*F* = 0.43, *P* = 0.730) or any ICN pairs (*P* > 0.1 for all pairs). Exploratory analyses testing differences between suicidality and non-suicidality groups within each diagnostic category independently were all found to be significant at *P* < 0.01 (full results in Supplementary Table 7 and Supplementary Fig. 3).

Analysis of suicidal versus non-suicidal groups also remain unchanged when only participants with a clinical diagnosis are included (controls removed) (Supplementary Tables 8 and 9).

### Association between connectivity and current clinical symptoms (DASS)

After controlling for multiple comparisons, there were no associations between depression, anxiety or stress symptoms and mean suicidality network connectivity (for anxiety, *r* = −0.049, *P* = 0.456; for depression, *r* = 0.139, *P* = 0.036; for stress, *r* = 0.096, *P* = 0.145) or mean connectivity between the ICN pairs (Supplementary Table 10). In addition, controlling for DASS when comparing suicidality and no-suicidality groups on extracted network connectivity and ICN does not change the significance of the results (Supplementary Tables 11–14).

## Discussion

The major finding of this study was that individuals who were recently suicidal had decreased connectivity within and between the VIS, SMN, and ventral attention/salience network, and between these networks and the DMN and DAN. We also observed lower connectivity between the VIS and SMN and the limbic network. Although these observed differences between people with and without suicidality are consistent with many previous reports^19^, this study advances our understanding of the neural underpinnings of suicidality in two key ways. First, this study adopted a transdiagnostic approach, specifically including those with a mood, anxiety or stress disorder. Second, by profiling the connectome of a very large sample, this study improved the robustness of our analyses relative to nearly all previous studies of neural biomarkers of suicidality.

The hypoconnectivity within the VIS and between this network and the SMN, DMN and ventral attention/salience network is interesting because of evidence regarding the role of visual imagery in suicidality. Studies have shown that suicidality involves vivid suicide-related imagery, including ‘flash-forwards’ of possible future suicide attempts^20,21^. There is convergent indirect evidence of the role of VISs being implicated in suicidality. One study found decreased occipital volume in those who attempted suicide^22^. There is also evidence of low-frequency fluctuations in the middle occipital gyrus in people who are suicidal^23^. Given the role of VISs in imagery, it is possible that the aberrant connectivity within and between the VIS and other large-scale networks may play a role in how visual imagery interacts with somatosensory, salience and introspective processes involved in suicidality^21^.

The observed decreased connectivity within the SMN accords with evidence of hypoconnectivity in this network in a range of psychiatric disorders, including depression^24^, bipolar disorder^25^, and PTSD^26^ and in survivors of childhood abuse^27^. Further, diminished connectivity with the SMN has been shown in transdiagnostic studies, suggesting this may reflect an underlying characteristic of general psychopathology^28^. There is increasing recognition that the sensorimotor system is critical for emotional functioning because the sensorimotor system underpins communicating emotions between individuals, and this has a direct impact on an individual’s emotional experiences^29^. This perspective is relevant to suicidality, such that people with a history of suicidality are highly sensitive to certain affective stimuli, especially when associated with self-harm^30^. This may also explain our finding of connectivity differences related to the ventral attention/salience brain network (and particularly for the insula)—a network of brain regions associated with evaluation and processing of internal and external stimuli to facilitate appropriate behavioral responses. Lower within-network connectivity in this network has been associated with suicidal history^15,31^.

The observed altered functional hypoconnectivity between both the VIS and SMN with the limbic network in participants who are suicidal is in line with findings of hypoconnectivity between the limbic network and a range of other neural regions in patients with depression and in those with a history of suicidal attempts^32,33^. This accords with meta-analytic evidence that suicidality is associated with functional differences in limbic, cognitive and default mode networks^34^. Interestingly, one study reported increased connectivity between the SMN and the insula; however, this study comprised a small sample and focused only on patients who were depressed and had a history of suicidality^35^. There is much evidence of the role of the limbic system in suicidality, which is expected given its function in emotion processing^36^. Although previous studies have identified the role of the amygdala in suicidal behavior^37,38^, we did not find any connections related to the amygdala to be altered in our analysis. The limbic connections identified in our study involved the hippocampus, temporal pole and OFC (Supplementary Table 2). Nevertheless, this finding underscores the importance of the connectivity between limbic and visual–somatomotor networks because these regions integrate emotional and behavioral responses, which are highly relevant to suicidality.

We also observed decreased connectivity between the DMN and the DAN, ventral attention network and the SMN. Suicidality has been associated with hypoconnectivity within the DMN^31^. Although we did not observe within-network hypoconnectivity for the DMN, we replicate previous findings of internetwork connectivity between the DMN and a range of networks, particularly the DAN^15,39^ and the salience (ventral attention) network^40^.

The DMN has been implicated in self-focused thinking about one’s past and future^41,42^. Accordingly, the aberrant connectivity findings in the DMN that have been noted in suicidality has been interpreted as the result of rumination and negative self-focus^43^. The observation in our study of hypoconnectivity between the DMN and SMN in participants who are suicidal may reflect these networks’ respective roles in ruminative self-focus and communicating of emotions, both of which can be relevant to suicidality.

In terms of study limitations, we recognize that our assessment of suicidality was limited to ideation, plans and attempts in the past month. This approach was taken to achieve a measure of very recent or current suicidal tendencies; however, it is possible that some participants identified as suicidal may not have been actively suicidal at the time of their testing. Future studies could recruit participants who are currently suicidal, not currently suicidal but have a history of suicidality and with no history of suicidality. It would also be interesting to evaluate brain connectivity differences in those with active (for example, have a plan) versus passive (for example, wish they were dead) ideations. Adequately powered trials with sufficient participants in each of these groups would permit a more nuanced understanding of the functional connectomes of suicidality. Further, although this sample size was very large relative to all previous investigations of connectivity in people who are suicidal, the sample sizes of a number of specific clinical groupings was not large (<30) and this precludes robust analysis of differential connectomes that may underlie suicidality in different psychiatric disorders. Although we evaluated across mood, anxiety and stress disorders, our cohort did not include individuals with other main psychiatric diagnoses, such as schizophrenia, psychosis, bipolar and attention deficit hyperactivity disorders. Including a broader diagnostic cohort would have made our analyses truly transdiagnostic. We also note that the study used intrinsic connectivity derived from task fMRI (both block and event-related designs). Previous work has suggested that although block task-fMRI designs are suited for intrinsic connectivity, results should be interpreted with caution when using intrinsic connectivity derived from event-related tasks^14^. Our previous work^44^ has shown consistent intrinsic connectivity across the tasks used in this study; however, caution should be applied when comparing results with resting-state studies or studies that use intrinsic data derived from other fMRI tasks.

In summary, this study represents a substantial advance in understanding the functional connectome of suicidality by examining neural connectivity in a very large sample and by adopting a transdiagnostic approach across disorders of mood, anxiety and stress to overcome confounds with specific clinical diagnoses. The pattern of findings highlighting the role of the SMN and VIS, and its connections with the default mode, salience and limbic networks points to neural underpinnings of key processes that are well documented in suicidal tendencies.

## Methods

### Participants

The Western Sydney Area Health Service Ethics Committee approved all procedures involved in this study, and all participants provided written informed consent. Participants (aged 18–65 years) were recruited at the Westmead Institute for Medical Research with the goal of obtaining a sample of people with anxiety or depressive disorders along with healthy controls. Participants were recruited from advertising and referral from clinicians to participate in research on neural markers of psychological well-being as part of two studies, the details of which are provided in Supplementary Table 1. Participants were screened for mental disorders using the Mini International Neuropsychiatric Interview, a structured interview with *Diagnostic and Statistical Manual of Mental Disorders 4th Edition*criteria^45^. Dimensional measures of anxiety and depression were assessed using the self-reported DASS^46^. Suicidality was measured using the Mini International Neuropsychiatric Interview (MINI-Plus) suicidal scale. All participants, irrespective of current diagnoses or symptoms, were classified as suicidal if in the past month they (1) wished they were dead, (2) wished to harm themselves, (3) contemplated suicide, (4) had a suicide plan, or (5) had attempted suicide; in addition, those who had attempted suicide in their lifetime were also classified as suicidal. Suicidal ideation was included in this definition because it is repeatedly shown to be a key predictor of suicide attempts^47,48^. Remaining individuals who reported no current or past suicidal symptoms or ideations were categorized into the non-suicidal group. There were initially 660 participants recruited; however, of these, 5 were missing relevant clinical data, 35 failed to complete the MRI, 39 displayed excessive movement within the MRI and 2 had artifacts or registration issues in the scanner. Accordingly, 579 participants were available for analysis, of whom 309 were women (53.4%). The mean ± s.d. age of the sample was 35.5 ± 12.5 years. Groups were compared on demographic and clinical characteristics using either two-sided *t-*tests (continuous measures) or Pearson’s chi-squared tests (categorical measures) (Table 1) run using R (v.4.0.3).

### fMRI acquisition and preprocessing

All participants completed MRI scans on a single 3T GE Signa Twinspeed HDxT MR Scanner (GE Healthcare) using an 8 channel phased-array head coil. Five fMRI tasks and one T1-weighted structural image were collected from each participant. For the functional scans, an echo planar imaging sequence with the following parameters was used: repetition time (TR) = 2,500 ms, echo time (TE) = 27.5 ms, matrix = 64 × 64, field of view (FOV) = 24 cm, flip angle = 90° and 120 volumes. The total scan time for each sequence was 5 min and 8 s (for 4 dummy scans), and each whole-brain volume consisted of 40 oblique axial slices that were 3.5 mm thick. Three dummy scans were acquired preceding each acquisition to allow magnetization to stabilize to steady state. The functional tasks involved the go/no-go (response inhibition), auditory oddball, working memory (n-back), facial emotion processing and emotional reappraisal tasks, and have been described previously^49^.

Structural images were acquired in the sagittal plane using a 3-dimensional spoiled gradient recalled (3D-SPGR) sequence with the following parameters: FOV = 256 mm, TR = 8.3 ms, TE = 3.2 ms, flip angle = 11°, inversion time (TI) = 500 ms, number of excitations (NEX) = 1, array spatial sensitivity encoding technique (ASSET) = 1.5, frequency direction = superior/inferior, 256 × 256 matrix, 180 contiguous sagittal slices and 1 mm isotropic voxels. The structural scan was used to normalize the functional data.

As no resting-state fMRI data were available, intrinsic functional connectivity data were extracted by concatenating the residual time series of the functional scans. This method has been validated previously^14,49^. Using the general linear model framework separately for each task, the blood-oxygen-level dependent (BOLD) responses were modeled for each experimental condition within each fMRI task. Physiological noise was removed by including covariates in the model that reflected the mean signal time course derived from cerebrospinal fluid and white matter masks, and temporal masks were used for movement. The final resting-state images were derived by modeling and regressing out the variance in BOLD signal associated with each of the stimuli in the tasks as a covariate. A band-pass filter (0.009 Hz < frequency < 0.08 Hz) was then applied to the signal, resulting in a time series of 600 volumes from which intrinsic resting-state connectivity can be measured. Intrinsic connectivity has previously been shown to show very similar patterns of functional connectivity compared with pure resting-state data^44,50^.

Volumes with a fractional displacement (FD) of 0.3 mm or higher or those with a signal scale intensity difference of more than 10 between volumes were excluded as movement outliers. The volumes on either side of the outlier were also excluded. The Volterra expansion of 24 realignment parameters was also modeled for each task^51^ and participants whose total mean FD exceeded 0.2 were excluded.

### Generating functional connectomes

Each participant’s average time series was extracted from 400 cortical regions^52^ and 36 subcortical regions^53^ that were clustered into 7 ICNs. The BOLD time series from the intrinsic connectivity data was then extracted for each parcel and correlated pairwise with all other parcels. Finally, a Fisher *Z*-transform was applied to produce a 436 × 436 functional correlation matrix (that is, functional connectome) for every participant.

ICNs were defined as per the Schaefer parcellation or as belonging to the subcortical network. The labeled networks were the DMN, DAN, ventral attention network, cognitive control network, VIS, SMN and limbic network or subcortex.

### Connectome differences between participants who were suicidal and those who were not suicidal

Functional connectomes were analyzed to test differences in whole-brain intrinsic connectivity between participants who were suicidal and those who were not suicidal using the NBS^54^. The NBS is a validated, non-parametric method that accounts for multiple comparisons by basing hypothesis tests on interconnected subnetworks rather than on individual connections. In this approach, *t-*tests are used to discover links between parcels that differ between groups. Connections that pass the *t*-statistic threshold are combined to form networks. Each network is tested for significance by comparing it to networks formed through random permutation, resulting in family-wise-corrected *P* values. In this analysis, each participant’s functional connectivity matrix (generated as above) was submitted to the NBS toolbox along with a design matrix that included a categorical variable representing participant group (suicidal or non-suicidal), used to test group difference across the connectome in both directions (suicidal > non-suicidal and non-suicidal > suicidal). Clinical diagnosis (individual diagnostic categories defined in Table 1), age in years, gender and years of education were also included in the design matrix as covariates of no interest in the NBS analysis due to a known association with functional connectivity and/or as they were different between the groups. A *t*-statistic threshold of 3.3 was used, which is equivalent to a *P* value of less than 0.001 and a Cohen’s effect size of greater than 0.3. Networks that survived a family-wise corrected *P* < 0.05 were regarded as significant. Details of the NBS method are described further in Zalesky et al., 2010^54^.

Functional connectivity values were extracted for each connection within the NBS-identified network. To assist in describing and interpreting our results, connections to parcels within ICNs were grouped together and mean intranetwork and internetwork connectivity was evaluated. Further post hoc analyses were done as described below in R using extracted connectivity data from the substantial network identified in NBS averaged across all connections to create a total network mean and averaged across connections for each intrinsic network connectivity pair. A Bonferroni-corrected *P* < 0.05 was used to account for the number of intrinsic connectivity pairs tested in all post hoc analyses.

### Post hoc analyses

#### Differences in connectivity due to current suicidality

Using the extracted connectivity measures above, we tested whether differences in connectivity observed in the main analysis were impacted by current suicidality (that is, suicidality in the past month) as opposed to previous suicidality (lifetime). Participants were divided into those who were currently suicidal (including those with a previous attempt), previously suicidal (lifetime attempt but no current symptoms) and non-suicidal (independent variable) and an analysis of covariance (ANCOVA) was run comparing these three suicidality groups, while controlling for age, gender, clinical diagnosis and years of education. We also subdivided the currently suicidal group further into those with and without a previous attempt and repeated the above comparisons to rule out the impact of a previous attempt on the currently suicidal group.

#### Differences in connectivity due to previous suicide attempts versus only ideation

To test the impact of a suicide attempt (as opposed to suicidal ideations alone) on connectivity differences, the sample was also limited to include only participants with suicidality, divided into participants who had attempted suicide (*n* = 103) and those who had not attempted suicide (*n* = 135). An ANCOVA was run to test connectivity differences between those who attempted suicide and those who had not (independent variable), controlling for clinical diagnosis, age, gender and years of education.

#### Differences in connectivity due to clinical diagnosis

In this analysis, we tested the effects of current clinical diagnosis on suicidality-related connectivity differences. Considering there were substantial differences between the rate of clinical diagnosis across the suicidal and non-suicidal groups but small cell sizes of groups with the same specific clinical diagnosis, we combined the clinical cohorts into four diagnostic categories: depression (MDDs), anxiety disorders (general anxiety disorders, social anxiety disorders and panic disorder), stress disorders (PTSD and complex PTSD) and controls (healthy controls, grief controls, trauma controls and those with mTBI). We evaluated in an analysis of variance (dependent variables: main effects and interaction of suicidality and diagnostic category) if there were significant suicidality × diagnostic category interactions with functional connectivity and ran exploratory *t*-tests to evaluate differences between participants who were non-suicidal and those who were suicidal for each diagnostic cohort to determine any clinical specificity of the suicidality network identified, while controlling for age, gender, clinical diagnosis and years of education as covariates. Further, considering the large number of participants without a diagnosis in the non-suicidal group (healthy controls, trauma controls, grief controls and those with mTBI), we also performed a supplementary analysis removing these participants to confirm the identified network was not influenced by over-representation of healthy controls in the non-suicidality group.

#### Association between connectivity and current clinical symptoms (DASS)

Finally, we ran bivariate correlations between suicidality-related functional connectivity using the extracted total network mean and means of ICN pairs and current clinical symptoms (DASS scores) to evaluate whether the degree of connectivity of the suicidality network was associated with current symptomatology. We also performed a supplementary ANCOVA between the suicidal and non-suicidal groups (independent variable) for extracted mean connectivity of the identified network controlling for DASS measures to assess if current clinical symptoms impacted observed differences in connectivity.

### Reporting summary

Further information on research design is available in the Nature Portfolio Reporting Summary linked to this article.

## Supplementary information

Supplementary Information Supplementary Tables 1–14 and Figs. 1 and 2.

Reporting Summary

## Data Availability

The data used in this study can be made available from the corresponding author upon reasonable request. Due to privacy and ethical requirements the data are not publicly available.
