# Supplementary material for: The neural connectome of suicidality in adults with mood and anxiety disorders
Source: Nat Ment Health. 2024 Oct 4;2(11):1342–9. doi: 10.1038/s44220-024-00325-y (PMC11540851; doi:10.1038/s44220-024-00325-y)
Supplement: Supplementary file 1 — Supplementary Tables 1–14 and Figs. 1 and 2. [file 44220_2024_325_MOESM1_ESM.pdf]

nature mental health

Supplementary information

https://doi.org/10.1038/s44220-024-00325-y

The neural connectome of suicidality in adults with mood and anxiety disorders

In the format provided by the authors and unedited

Table S1. Summary of contributing studies

Study Name	Number of Participants in Analysis	Study Criteria	Data collected	References
International Study to Predict Optimized Treatment for Depression (iSPOT-D)	292	Inclusion:<br>• Age 18-65<br>• Fluent and literate in English<br>• Provide written informed consent.<br>• HRSD17 ≤ 16<br>• Meets DSM-IV criteria for single or recurrent nonpsychotic MDD (using MINI plus)<br>Exclusion:<br>• Score of ≥ 8 on Section C of MINI Plus (actively suicidal)<br>• History of bipolar disorder, schizophrenia, schizoaffective disorder, or psychosis<br>• Current primary diagnosis of anorexia/bulimia, OCD, PTSD<br>• Known contraindication to escitalopram, sertraline, venlafaxine or previous treatment failure at highest dose<br>• Taking other contraindicated medications<br>• Use of non-protocol antidepressant or CNS that cannot be washed out<br>• General medical condition contraindicative to antidepressant treatment or protocol<br>• Substance dependence	Interview:<br>• **Mini International Neuropsychiatric Interview, a structured interview with DSM-IV criteria.**<br>• 17-item & 21-item Hamilton Rating Scale for Depression (HRSD17)<br>Web Self-report (WebQ):<br>• **Demographic Medical History**<br>• **Depression, Anxiety and Stress Scale (DASS).**<br>• **Early Life Stress Questionnaire (ELSQ)**<br>• 16-item Quick Inventory of Depressive Symptomatology ($QIDS_{16}$)<br>MRI (3.0T GE SignaTwinspeed, Westmead):<br>• **T1 SPGR**<br>• DTI<br>• **fMRI tasks:** Oddball, CPT, Go-NoGo, unmasked conscious emotion	Williams, L.M., Rush, A.J., Koslow, S.H. *et al.* International Study to Predict Optimized Treatment for Depression (iSPOT-D), a randomized clinical trial: rationale and protocol. *Trials* **12**, 4 (2011). https://doi.org/10.1186/1745-6215-12-4<br><br>Grieve, S.M., Korgaonkar, M.S., Etkin, A. *et al.* Brain imaging predictors and the international study to predict optimized treatment for depression: study protocol for a randomized controlled trial. *Trials* **14**, 224 (2013). https://doi.org/10.1186/1745-6215-14-224<br><br>ClinicalTrials.gov ID: NCT00693849;

		• Brain injury or blow to head that result in loss of consciousness for greater than five minutes.<br>• Severe impediment to vision, hearing or hand movement that is likely to interfere to comprehension or completion of assessment.<br>• Pregnant or breast-feeding<br>• Participant in investigational study within four months that could impact MDD symptoms.	processing, masked non-conscious emotion processing	
NHMRC Centre for Clinical Research Excellence in Anxiety Disorders (CCRE)	287	Inclusion:<br>• Age 18-65<br>• Fluent and literate in English<br>• Provide written informed consent.<br>• Meets DSM-IV criteria for mood or anxiety disorder (using MINI 5.0) or healthy control.<br>• Medication permitted as long as dosage had been stable for previous two months.<br>Exclusion:<br>• History of bipolar disorder, schizophrenia, schizoaffective disorder, or psychosis<br>• General medical condition contraindicative to antidepressant treatment or protocol<br>• Substance dependence	Interview:<br>• **Mini International Neuropsychiatric Interview, a structured interview with DSM-IV criteria.**<br>• Beck Depression Investory-2 (BDI)<br>• Clinician Administered Posttraumatic Stress Disorder Scale (CAPS)<br>Web Self-report (WebQ):<br>• **Demographic Medical History**<br>• **Depression, Anxiety and Stress Scale (DASS).**<br>• **Early Life Stress Questionnaire (ELSQ)**	R. A. Bryant *et al.*, Reappraisal-related neural predictors of treatment response to cognitive behavior therapy for post-traumatic stress disorder. *Psychol Med* **51**, 2454-2464 (2021). https://doi.org/10.1017/S0033291720001129

		• Brain injury or blow to head that result in loss of consciousness for greater than five minutes.<br>• Severe impediment to vision, hearing or hand movement that is likely to interfere to comprehension or completion of assessment.<br>• Pregnant	MRI (3.0T GE SignaTwinspeed, Westmead):<br>• **T1 SPGR**<br>• DTI<br>• **fMRI tasks:** Emotional Reappriasal Task x2, Go-NoGo, unmasked conscious emotion processing, masked non-conscious emotion processing.	

*Common measures used in this analysis shown in **bold***

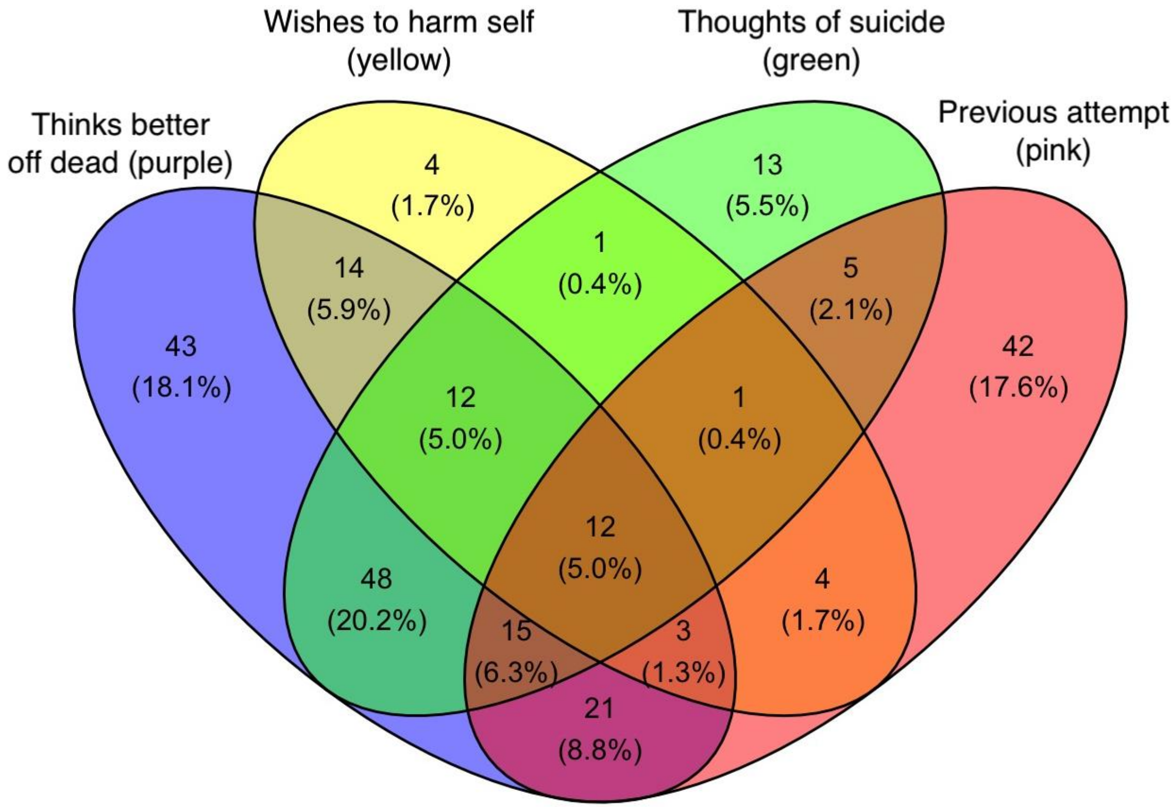

Figure S1. Venn diagram capturing responses of participants to the suicidal scale of the MINI. Responses included: 1. Thinks better off dead (purple, n=168); 2. wishes to harm themselves (yellow, n=51); 3. Thoughts of suicide (green, n=107, this included those who responded yes to 4. Suicide plan (n=6) and 5. recent suicide attempt (item 5, n=2)); 6) Previous suicide attempt (pink, n = 103)

Table S2. Summary of number and direction of edges within or between ICNs that decreased in suicidal participants as identified with NBS

NETWORK	TOTAL CONNECTIONS	% Connections*	NEGATIVE CONNECTIONS	POSITIVE CONNECTIONS
DMN-VAN	2	1.40	1	1
DMN-DAN	1	0.70	-	1
DMN-SMN	30	20.98	-	30
DMN-VIS	4	2.80	-	4
LIMBIC-SMN	6	4.20	1	5
LIMBIC-VIS	4	2.80	1	3
VAN-VIS	1	0.70	-	1
VAN-VAN	1	0.70	-	1
VAN-SMN	31	21.68	-	31
DAN-VIS	2	0.00	-	2
SMN-VIS	32	0.00	-	32
SMN-SMN	27	1.40	-	27
VIS-VIS	2	22.38	-	2

*Percentage of total connections (143) for that network relationship

Table S3. Complete List of Significant Connections between Suicidal and Non-Suicidal Individuals identified with NBS (Threshold 3.3)

	Region1	MNI (X)	MNI (Y)	MIN (Z)	Network	Region2	MNI (X)	MNI (Y)	MIN (Z)	Network	TestStat
						**Visual to Visual**					
1	8_Vis_8_L	-46	-70	-8	Vis	25_Vis_25_L	-2	-84	24	Vis	3.35
2	14_Vis_14_L	-42	-86	-4	Vis	219_Vis_19_R	10	-74	8	Vis	3.34
						**Visual to Somatomotor**					
1	25_Vis_25_L	-2	-84	24	Vis	242_SomMot_12_R	60	0	10	SomMot	4.18
2	219_Vis_19_R	10	-74	8	Vis	242_SomMot_12_R	60	0	10	SomMot	3.93
3	219_Vis_19_R	10	-74	8	Vis	246_SomMot_16_R	60	-6	26	SomMot	3.85
4	225_Vis_25_R	4	-80	24	Vis	242_SomMot_12_R	60	0	10	SomMot	3.84
5	21_Vis_21_L	-8	-74	10	Vis	242_SomMot_12_R	60	0	10	SomMot	3.83
6	13_Vis_13_L	-14	-44	-6	Vis	55_SomMot_24_L	-4	-24	56	SomMot	3.79
7	4_Vis_4_L	-24	-54	-8	Vis	235_SomMot_5_R	38	-8	14	SomMot	3.76
8	11_Vis_11_L	-6	-76	-6	Vis	242_SomMot_12_R	60	0	10	SomMot	3.75
9	5_Vis_5_L	-24	-72	-10	Vis	242_SomMot_12_R	60	0	10	SomMot	3.7
10	4_Vis_4_L	-24	-54	-8	Vis	241_SomMot_11_R	50	-10	14	SomMot	3.64
11	10_Vis_10_L	-12	-62	-4	Vis	242_SomMot_12_R	60	0	10	SomMot	3.63
12	4_Vis_4_L	-24	-54	-8	Vis	242_SomMot_12_R	60	0	10	SomMot	3.6
13	219_Vis_19_R	10	-74	8	Vis	244_SomMot_14_R	62	-12	16	SomMot	3.56
14	21_Vis_21_L	-8	-74	10	Vis	246_SomMot_16_R	60	-6	26	SomMot	3.56
15	25_Vis_25_L	-2	-84	24	Vis	268_SomMot_38_R	6	-22	72	SomMot	3.56
16	25_Vis_25_L	-2	-84	24	Vis	64_SomMot_33_L	-4	-26	68	SomMot	3.53
17	11_Vis_11_L	-6	-76	-6	Vis	244_SomMot_14_R	62	-12	16	SomMot	3.51
18	13_Vis_13_L	-14	-44	-6	Vis	260_SomMot_30_R	4	-24	58	SomMot	3.5
19	22_Vis_22_L	-18	-64	6	Vis	242_SomMot_12_R	60	0	10	SomMot	3.49
20	225_Vis_25_R	4	-80	24	Vis	244_SomMot_14_R	62	-12	16	SomMot	3.49
21	13_Vis_13_L	-14	-44	-6	Vis	50_SomMot_19_L	-40	-14	48	SomMot	3.44
22	10_Vis_10_L	-12	-62	-4	Vis	35_SomMot_4_L	-38	-8	12	SomMot	3.41
23	21_Vis_21_L	-8	-74	10	Vis	244_SomMot_14_R	62	-12	16	SomMot	3.4
24	13_Vis_13_L	-14	-44	-6	Vis	54_SomMot_23_L	-8	-38	54	SomMot	3.38
25	25_Vis_25_L	-2	-84	24	Vis	244_SomMot_14_R	62	-12	16	SomMot	3.36
26	209_Vis_9_R	50	-64	-10	Vis	250_SomMot_20_R	12	-18	42	SomMot	3.34
27	25_Vis_25_L	-2	-84	24	Vis	63_SomMot_32_L	-8	-42	70	SomMot	3.34

28	10_Vis_10_L	-12	-62	-4	Vis	244_SomMot_14_R	62	-12	16	SomMot	3.31
29	22_Vis_22_L	-18	-64	6	Vis	246_SomMot_16_R	60	-6	26	SomMot	3.31
30	211_Vis_11_R	10	-72	-6	Vis	242_SomMot_12_R	60	0	10	SomMot	3.3
31	206_Vis_6_R	24	-74	-10	Vis	244_SomMot_14_R	62	-12	16	SomMot	3.3
32	53_SomMot_22_L	-38	-24	52	SomMot	216_Vis_16_R	48	-66	4	Vis	3.52
**Visual to Salience/Ventral Attention**											
1	4_Vis_4_L	-24	-54	-8	Vis	305_SalVentAttn_FrOper_4_R	40	-2	6	SalVentAttn	3.57
**Visual to Limbic**											
1	28_Vis_28_L	-32	-84	26	Vis	321_Limbic_OFC_3_R	8	46	-24	Limbic	3.46
2	211_Vis_11_R	10	-72	-6	Vis	331_Limbic_TempPole_7_R	22	-18	-28	Limbic	3.42
3	21_Vis_21_L	-8	-74	10	Vis	126_Limbic_TempPole_8_L	-20	-20	-26	Limbic	3.41
4	28_Vis_28_L	-32	-84	26	Vis	117_Limbic_OFC_4_L	-4	24	-20	Limbic	3.35
**Visual to Default Mode**											
1	25_Vis_25_L	-2	-84	24	Vis	373_Default_Temp_7_R	64	-38	0	Default	3.55
2	25_Vis_25_L	-2	-84	24	Vis	158_Default_Temp_10_L	-52	-44	4	Default	3.39
3	25_Vis_25_L	-2	-84	24	Vis	371_Default_Temp_5_R	64	-24	-8	Default	3.38
4	225_Vis_25_R	4	-80	24	Vis	373_Default_Temp_7_R	64	-38	0	Default	3.32
**Somatomotor to Somatomotor**											
1	242_SomMot_12_R	60	0	10	SomMot	250_SomMot_20_R	12	-18	42	SomMot	3.91
2	64_SomMot_33_L	-4	-26	68	SomMot	250_SomMot_20_R	12	-18	42	SomMot	3.83
3	42_SomMot_11_L	-62	-18	20	SomMot	270_SomMot_40_R	12	-32	76	SomMot	3.76
4	35_SomMot_4_L	-38	-8	12	SomMot	63_SomMot_32_L	-8	-42	70	SomMot	3.76
5	231_SomMot_1_R	52	4	-6	SomMot	252_SomMot_22_R	44	-10	48	SomMot	3.71
6	35_SomMot_4_L	-38	-8	12	SomMot	68_SomMot_37_L	-12	-26	74	SomMot	3.71
7	56_SomMot_25_L	-4	-8	60	SomMot	237_SomMot_7_R	60	-24	10	SomMot	3.67
8	42_SomMot_11_L	-62	-18	20	SomMot	269_SomMot_39_R	16	-18	74	SomMot	3.66
9	233_SomMot_3_R	54	-14	6	SomMot	254_SomMot_24_R	6	-10	52	SomMot	3.63
10	56_SomMot_25_L	-4	-8	60	SomMot	238_SomMot_8_R	64	-34	10	SomMot	3.62
11	237_SomMot_7_R	60	-24	10	SomMot	262_SomMot_32_R	10	-40	68	SomMot	3.61
12	250_SomMot_20_R	12	-18	42	SomMot	268_SomMot_38_R	6	-22	72	SomMot	3.53

13	231_SomMot_1_R	52	4	-6	SomMot	254_SomMot_24_R	6	-10	52	SomMot	3.51
14	42_SomMot_11_L	-62	-18	20	SomMot	263_SomMot_33_R	22	-24	66	SomMot	3.5
15	234_SomMot_4_R	40	-20	4	SomMot	263_SomMot_33_R	22	-24	66	SomMot	3.47
16	234_SomMot_4_R	40	-20	4	SomMot	264_SomMot_34_R	22	-28	68	SomMot	3.47
17	250_SomMot_20_R	12	-18	42	SomMot	263_SomMot_33_R	22	-24	66	SomMot	3.44
18	234_SomMot_4_R	40	-20	4	SomMot	249_SomMot_19_R	54	-16	40	SomMot	3.43
19	64_SomMot_33_L	-4	-26	68	SomMot	238_SomMot_8_R	64	-34	10	SomMot	3.41
20	242_SomMot_12_R	60	0	10	SomMot	249_SomMot_19_R	54	-16	40	SomMot	3.41
21	246_SomMot_16_R	60	-6	26	SomMot	250_SomMot_20_R	12	-18	42	SomMot	3.41
22	35_SomMot_4_L	-38	-8	12	SomMot	64_SomMot_33_L	-4	-26	68	SomMot	3.41
23	55_SomMot_24_L	-4	-24	56	SomMot	232_SomMot_2_R	62	-18	0	SomMot	3.4
24	238_SomMot_8_R	64	-34	10	SomMot	268_SomMot_38_R	6	-22	72	SomMot	3.38
25	33_SomMot_2_L	-56	-22	8	SomMot	238_SomMot_8_R	64	-34	10	SomMot	3.36
26	42_SomMot_11_L	-62	-18	20	SomMot	250_SomMot_20_R	12	-18	42	SomMot	3.32
27	233_SomMot_3_R	54	-14	6	SomMot	250_SomMot_20_R	12	-18	42	SomMot	3.3
					**Somatomotor to Salience/Ventral Attention**						
1	251_SomMot_21_R	52	-12	50	SomMot	305_SalVentAttn_FrOper_4_R	40	-2	6	SalVentAttn	4.07
2	252_SomMot_22_R	44	-10	48	SomMot	308_SalVentAttn_FrOper_7_R	48	4	4	SalVentAttn	4.06
3	260_SomMot_30_R	4	-24	58	SomMot	304_SalVentAttn_FrOper_3_R	40	-10	-4	SalVentAttn	3.92
4	249_SomMot_19_R	54	-16	40	SomMot	305_SalVentAttn_FrOper_4_R	40	-2	6	SalVentAttn	3.88
5	55_SomMot_24_L	-4	-24	56	SomMot	316_SalVentAttn_Med_6_R	10	-32	50	SalVentAttn	3.86
6	54_SomMot_23_L	-8	-38	54	SomMot	305_SalVentAttn_FrOper_4_R	40	-2	6	SalVentAttn	3.71
7	262_SomMot_32_R	10	-40	68	SomMot	305_SalVentAttn_FrOper_4_R	40	-2	6	SalVentAttn	3.62
8	255_SomMot_25_R	44	-22	54	SomMot	305_SalVentAttn_FrOper_4_R	40	-2	6	SalVentAttn	3.59
9	248_SomMot_18_R	52	-6	38	SomMot	305_SalVentAttn_FrOper_4_R	40	-2	6	SalVentAttn	3.58
10	64_SomMot_33_L	-4	-26	68	SomMot	100_SalVentAttn_FrOper_4_L	-40	-14	-2	SalVentAttn	3.56
11	252_SomMot_22_R	44	-10	48	SomMot	305_SalVentAttn_FrOper_4_R	40	-2	6	SalVentAttn	3.56
12	254_SomMot_24_R	6	-10	52	SomMot	305_SalVentAttn_FrOper_4_R	40	-2	6	SalVentAttn	3.56
13	263_SomMot_33_R	22	-24	66	SomMot	304_SalVentAttn_FrOper_3_R	40	-10	-4	SalVentAttn	3.54

14	55_SomMot_24_L	-4	-24	56	SomMot	304_SalVentAttn_FrOper_3_R	40	-10	-4	SalVentAttn	3.52
15	259_SomMot_29_R	34	-28	62	SomMot	305_SalVentAttn_FrOper_4_R	40	-2	6	SalVentAttn	3.51
16	55_SomMot_24_L	-4	-24	56	SomMot	315_SalVentAttn_Med_5_R	10	-44	54	SalVentAttn	3.49
17	264_SomMot_34_R	22	-28	68	SomMot	305_SalVentAttn_FrOper_4_R	40	-2	6	SalVentAttn	3.47
18	264_SomMot_34_R	22	-28	68	SomMot	304_SalVentAttn_FrOper_3_R	40	-10	-4	SalVentAttn	3.46
19	254_SomMot_24_R	6	-10	52	SomMot	304_SalVentAttn_FrOper_3_R	40	-10	-4	SalVentAttn	3.44
20	260_SomMot_30_R	4	-24	58	SomMot	305_SalVentAttn_FrOper_4_R	40	-2	6	SalVentAttn	3.42
21	257_SomMot_27_R	32	-34	64	SomMot	305_SalVentAttn_FrOper_4_R	40	-2	6	SalVentAttn	3.42
22	263_SomMot_33_R	22	-24	66	SomMot	305_SalVentAttn_FrOper_4_R	40	-2	6	SalVentAttn	3.4
23	55_SomMot_24_L	-4	-24	56	SomMot	111_SalVentAttn_Med_5_L	-12	-42	48	SalVentAttn	3.39
24	49_SomMot_18_L	-8	-16	48	SomMot	304_SalVentAttn_FrOper_3_R	40	-10	-4	SalVentAttn	3.34
25	260_SomMot_30_R	4	-24	58	SomMot	316_SalVentAttn_Med_6_R	10	-32	50	SalVentAttn	3.33
26	55_SomMot_24_L	-4	-24	56	SomMot	100_SalVentAttn_FrOper_4_L	-40	-14	-2	SalVentAttn	3.32
27	63_SomMot_32_L	-8	-42	70	SomMot	305_SalVentAttn_FrOper_4_R	40	-2	6	SalVentAttn	3.31
28	100_SalVentAttn_FrOper_4_L	-40	-14	-2	SalVentAttn	260_SomMot_30_R	4	-24	58	SomMot	3.63
29	111_SalVentAttn_Med_5_L	-12	-42	48	SalVentAttn	242_SomMot_12_R	60	0	10	SomMot	3.53
30	96_SalVentAttn_TempOcc_1_L	-58	-54	10	SalVentAttn	246_SomMot_16_R	60	-6	26	SomMot	3.43
31	94_SalVentAttn_ParOper_3_L	-62	-24	32	SalVentAttn	254_SomMot_24_R	6	-10	52	SomMot	3.41

Somatomotor to Limbic

1	254_SomMot_24_R	6	-10	52	SomMot	406_LIMBICHipp_R	22	-12	-20	Limbic	3.61
2	236_SomMot_6_R	34	-22	14	SomMot	331_Limbic_TempPole_7_R	22	-18	-28	Limbic	3.58
3	236_SomMot_6_R	34	-22	14	SomMot	328_Limbic_TempPole_4_R	40	-14	-32	Limbic	3.51
4	49_SomMot_18_L	-8	-16	48	SomMot	406_LIMBICHipp_R	22	-12	-20	Limbic	3.45
5	238_SomMot_8_R	64	-34	10	SomMot	321_Limbic_OFC_3_R	8	46	-24	Limbic	3.41

6	234_SomMot_4_R	40	-20	4	SomMot	331_Limbic_TempPole_7_R	22	-18	-28	Limbic	3.34
					**Salience/Ventral Attention to Salience/Ventral Attention**						
1	111_SalVentAttn_Med_5_L	-12	-42	48	SalVentAttn	305_SalVentAttn_FrOper_4_R	40	-2	6	SalVentAttn	3.53
					**Salience/Ventral Attention to Default Mode**						
1	96_SalVentAttn_TempOcc_1_L	-58	-54	10	SalVentAttn	170_Default_PFC_5_L	-46	32	-10	Default	3.49
2	100_SalVentAttn_FrOper_4_L	-40	-14	-2	SalVentAttn	189_Default_PFC_24_L	-6	10	64	Default	3.46
					**Dorsal attention to Visual**						
1	79_DorsAttn_Post_11_L	-38	-38	50	DorsAttn	216_Vis_16_R	48	-66	4	Vis	3.62
2	76_DorsAttn_Post_8_L	-46	-30	44	DorsAttn	216_Vis_16_R	48	-66	4	Vis	3.43
					**Dorsal attention to Default Mode**						
1	76_DorsAttn_Post_8_L	-46	-30	44	DorsAttn	374_Default_Temp_8_R	50	-34	2	Default	3.44
					**Default Mode to Somatomotor**						
1	158_Default_Temp_10_L	-52	-44	4	Default	254_SomMot_24_R	6	-10	52	SomMot	4.3
2	158_Default_Temp_10_L	-52	-44	4	Default	252_SomMot_22_R	44	-10	48	SomMot	3.7
3	152_Default_Temp_4_L	-56	-8	-14	Default	250_SomMot_20_R	12	-18	42	SomMot	3.67
4	152_Default_Temp_4_L	-56	-8	-14	Default	270_SomMot_40_R	12	-32	76	SomMot	3.6
5	158_Default_Temp_10_L	-52	-44	4	Default	260_SomMot_30_R	4	-24	58	SomMot	3.57
6	155_Default_Temp_7_L	-52	-22	-6	Default	248_SomMot_18_R	52	-6	38	SomMot	3.56
7	156_Default_Temp_8_L	-60	-12	-2	Default	252_SomMot_22_R	44	-10	48	SomMot	3.53
8	155_Default_Temp_7_L	-52	-22	-6	Default	254_SomMot_24_R	6	-10	52	SomMot	3.52
9	156_Default_Temp_8_L	-60	-12	-2	Default	248_SomMot_18_R	52	-6	38	SomMot	3.51
10	154_Default_Temp_6_L	-60	-34	-4	Default	250_SomMot_20_R	12	-18	42	SomMot	3.45
11	156_Default_Temp_8_L	-60	-12	-2	Default	251_SomMot_21_R	52	-12	50	SomMot	3.45
12	152_Default_Temp_4_L	-56	-8	-14	Default	254_SomMot_24_R	6	-10	52	SomMot	3.44
13	158_Default_Temp_10_L	-52	-44	4	Default	250_SomMot_20_R	12	-18	42	SomMot	3.39

14	158_Default_Temp_10_L	-52	-44	4	Default	251_SomMot_21_R	52	-12	50	SomMot	3.38
15	157_Default_Temp_9_L	-62	-32	6	Default	252_SomMot_22_R	44	-10	48	SomMot	3.37
16	154_Default_Temp_6_L	-60	-34	-4	Default	248_SomMot_18_R	52	-6	38	SomMot	3.32
17	56_SomMot_25_L	-4	-8	60	SomMot	158_Default_Temp_10_L	-52	-44	4	Default	3.79
18	254_SomMot_24_R	6	-10	52	SomMot	374_Default_Temp_8_R	50	-34	2	Default	3.7
19	250_SomMot_20_R	12	-18	42	SomMot	372_Default_Temp_6_R	48	-20	-8	Default	3.58
20	55_SomMot_24_L	-4	-24	56	SomMot	158_Default_Temp_10_L	-52	-44	4	Default	3.55
21	55_SomMot_24_L	-4	-24	56	SomMot	155_Default_Temp_7_L	-52	-22	-6	Default	3.53
22	53_SomMot_22_L	-38	-24	52	SomMot	158_Default_Temp_10_L	-52	-44	4	Default	3.53
23	49_SomMot_18_L	-8	-16	48	SomMot	158_Default_Temp_10_L	-52	-44	4	Default	3.46
24	68_SomMot_37_L	-12	-26	74	SomMot	152_Default_Temp_4_L	-56	-8	-14	Default	3.42
25	263_SomMot_33_R	22	-24	66	SomMot	374_Default_Temp_8_R	50	-34	2	Default	3.41
26	53_SomMot_22_L	-38	-24	52	SomMot	374_Default_Temp_8_R	50	-34	2	Default	3.38
27	49_SomMot_18_L	-8	-16	48	SomMot	372_Default_Temp_6_R	48	-20	-8	Default	3.36
28	260_SomMot_30_R	4	-24	58	SomMot	374_Default_Temp_8_R	50	-34	2	Default	3.36
29	250_SomMot_20_R	12	-18	42	SomMot	374_Default_Temp_8_R	50	-34	2	Default	3.36
30	55_SomMot_24_L	-4	-24	56	SomMot	374_Default_Temp_8_R	50	-34	2	Default	3.33

Table S4. Two-way ANCOVA Between Previous Attempters (n=42), Current (n=196; including previous attempts) and No Suicidality Participants (n=341) and post-hoc comparisons

	**ANCOVA**		**Previous - Current**		**None - Current**		**Previous -None**	
	F	*p*	*t*	*p*	*t*	*p*	*t*	*p*
Whole network	25.52	2.464e-11	-0.42	0.903	5.83	0.0001	-3.85	0.000319
DMN_VAN	11.58	0.0000118	-0.03	1.000	3.62	0.00112	-2.16	0.075
DMN_DAN	7.81	0.00045	0.35	0.931	4.04	0.00014	-2.03	0.102
DMN_SMN	14.45	0.00000072	0.32	0.944	4.52	0.000001	-2.35	0.048
DMN_VIS	6.99	0.0010042	-0.79	0.704	2.85	0.012	-2.45	0.037
LIMBIC_SMN	18.04	0.000000026	0.49	0.873	4.37	0.000001	-2.09	0.088
LIMBIC_VIS	11.92	0.0000085	0.41	0.911	3.97	0.00019053	-1.94	0.123
VAN_VAN	9.36	0.0001005	-1.31	0.379	2.72	0.018	-2.89	0.010
VAN_SMN	17.99	0.000000027	-0.84	0.674	5.29	0.000001	-3.94	0.000001
VAN_VIS	6.52	0.0015897	0.69	0.763	3.14	0.004829	-1.17	0.463
DAN_Vis	11.46	0.000013187	-1.45	0.307	3.47	0.0014710	-3.47	0.0014529
SMN_SMN	20.37	0.000000003	-0.58	0.825	4.90	0.000001	-3.46	0015938
SMN_VIS	16.07	0.0000002	-0.36	0.930	4.40	0.000001	-2.94	0.0087418
VIS_VIS	8.68	0.00019452	-0.23	0.971	3.80	0.000001	-2.46	0.036

Table S5. Two-way ANCOVA Between Previous Attempters (n=42), Current with Previous Attempt (n=61), Current only (n=135) and no suicidality (n=341)

Group tests	SumSq	MeanSq	F-value	P-value
4 groups	0.756	0.252	17.01	1.386e-10
Post-Hoc Tests	Estimate	Std. Error	T-value	P-value
Current&Prev vs Current Only	-0.004	0.020	-0.194	0.997
No Suicidality vs Current Only	0.071	0.014	5.182	<0.000001
Previous Attempt (no current) vs CurrentOnly	-0.009	0.022	-0.418	0.975
No Suicidality vs Current&Prev	0.075	0.019	3.936	<0.000001
Previous Attempt (no current) vs Current&Prev	-0.005	0.025	-0.222	0.996
Previous Attempt (no current) vs No Suicidality	-0.080	0.022	-3.737	0.00107

Table S6. Two-way ANCOVA Results for each of the ICN pairs comparing between attempters and non-attempters

	SumSq	MeanSq	F-value	P-value
Default_Sal_Mean	0.002	0.002	0.093	0.761
Default_DAN_Mean	0.006	0.006	0.136	0.713
Default_SomMot_Mean	0.035	0.035	1.578	0.210
Default_Vis_Mean	0.027	0.027	0.878	0.350
Limbic_SomMot_Mean	0.001	0.001	0.069	0.793
Limbic_Vis_Mean	0.001	0.001	0.087	0.768
Sal_Sal_Mean	0.129	0.129	2.720	0.100
Sal_SomMot_Mean	0.019	0.019	0.837	0.361
Sal_Vis_Mean	0.016	0.016	0.526	0.469
DAN_Vis_Mean	0.208	0.208	5.094	0.025
SomMot_Mean	0.022	0.022	1.044	0.308
SomMot_Vis_Mean	0.009	0.009	0.528	0.468
Vis_Vis_Mean	0.004	0.004	0.133	0.716

Table S7. Two-sided t-test between each of the diagnostic categories individual controlling for age, gender, years of education

	Network Mean Difference - Suicidal vs Not Suicidal				Number of participants	
	Sum Sq	Mean	F value	P value	No	Yes
DEPRESSION only	0.272	0.27224	19.519	0.00001	110	145
ANXIETY only	0.1323	0.1323	12.066	0.00098	31	32
STRESS only	0.1559	0.15587	8.926	0.00389	26	48
CONTROLS Only	0.1127	0.11273	6.864	0.00954	174	13

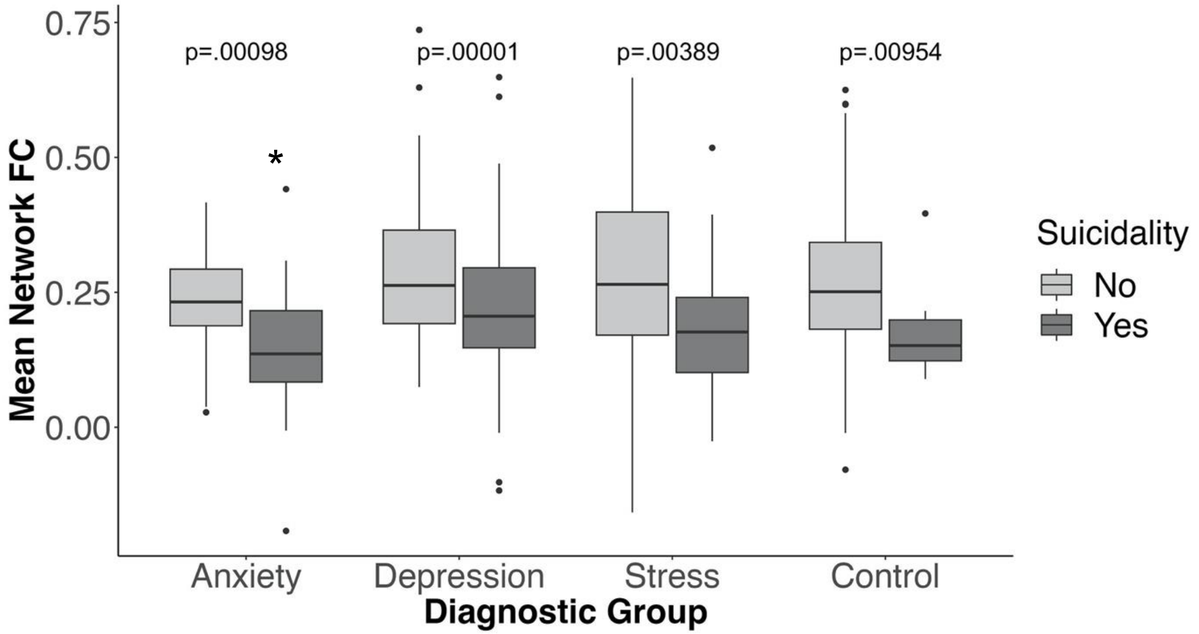

Figure S2. Two-sided t-test of difference in extracted mean network functional connectivity of identified suicidality network for each diagnostic category (anxiety, n=63 (suicidal, n=32; non-suicidal n=31), depression, n=255 (suicidal, n=145; non-suicidal n=110), stress, n=74 (suicidal, n=48; non-suicidal n=26), control, n=187 (suicidal, n=13; non-suicidal n=174)**.** Controlling for age, gender and years of education and Bonferroni corrected for multiple comparison (p<0.0125). Boxplot data are presented as median value (centre line) plus 1st and 3rd quartile respectively (box bounds) with whiskers representing minimum and maximum values respectively defined by 1st or 3rd quartile plus 1.5 times the interquartile range and single points representing outliers.

Table S8. Two-way ANOVA comparing suicidality vs no suicidality without healthy control participants

Network Mean - ANOVA Suicidality vs No Suicidality: No Controls						Original results: inc. controls	
	Df	SumSq	MeanSq	F-value	P-value	F-value	P-value
Sui.Yes	1	0.525	0.525	34.24	0.00000001033	47.027	0.00000000002

Table S9. Two-way ANOVA comparing mean intrinsic network functional connectivity in suicidality vs no suicidality groups without healthy control participants

Intrinsic Connectivity Networks – ANOVA Suicidality vs No Suicidality: No Controls						Original results: inc. controls	
Response Var – ICNs	df	sumsq	meansq	statistic	P-value	statistic	P-value
Default_Sal_Mean	1	0.440	0.440	17.30	0.0000393509	22.69	0.0000024111
Default_DAN_Mean	1	0.647	0.647	12.85	0.0003798110	14.74	0.0001368972
Default_SomMot_Mean	1	0.477	0.477	16.77	0.0000512801	26.83	0.0000003075
Default_Vis_Mean	1	0.159	0.159	5.30	0.0218996000	13.50	0.0002604958
Limbic_SomMot_Mean	1	0.322	0.322	24.70	0.0000010070	35.44	0.0000000046
Limbic_Vis_Mean	1	0.224	0.224	12.51	0.0004523352	22.23	0.0000030370
Sal_Sal_Mean	1	0.713	0.713	14.75	0.0001429160	15.41	0.0000968203
Sal_SomMot_Mean	1	0.817	0.817	30.00	0.0000000775	31.86	0.0000000260
Sal_Vis_Mean	1	0.272	0.272	7.42	0.0067417760	12.74	0.0003885090
DAN_Vis_Mean	1	0.671	0.671	14.28	0.0001818099	18.14	0.0000239975
SomMot_Mean	1	0.632	0.632	25.08	0.0000008330	37.15	0.0000000020
SomMot_Vis_Mean	1	0.398	0.398	21.46	0.0000049240	31.63	0.0000000291
Vis_Vis_Mean	1	0.362	0.362	10.55	0.0012611680	16.34	0.0000601528

Table S10. Two-sided Pearson's correlations between connectivity and each DASS symptom score within the suicidality group uncorrected for multiple comparisons

	**DASS Anxiety**		**DASS Depression**		**DASS Stress**	
	r	*p*	*r*	*p*	*r*	*p*
Whole network	-0.049	0.456	0.139	0.036*	0.096	0.145
DMN_VAN	0.016	0.808	0.004	0.957	0.045	0.499
DMN_DAN	-0.043	0.513	0.037	0.578	-0.022	0.736
DMN_SMN	-0.027	0.685	0.164	0.013	0.122	0.064
DMN_VIS	0.002	0.978	0.041	0.536	-0.048	0.463
LIMBIC_SMN	-0.065	0.321	-0.017	0.799	0.047	0.479
LIMBIC_VIS	-0.009	0.892	-0.006	0.923	-0.073	0.270
VAN_VIS	-0.015	0.817	0.088	0.182	0.061	0.359
VAN_VAN	-0.109	0.098	-0.047	0.481	0.002	0.976
VAN_SMN	-0.042	0.526	0.132	0.046*	0.110	0.094
DAN_VIS	-0.097	0.142	0.033	0.616	-0.019	0.771
SMN_VIS	-0.032	0.630	0.085	0.199	0.088	0.180
SMN_SMN	-0.053	0.425	0.122	0.066	0.057	0.390
VIS_VIS	-0.020	0.762	0.111	0.094	0.011	0.867

Table S11. Two-way ANOVA comparing suicidality vs no suicidality without healthy control participants and controlling for DASS

Network Mean - ANOVA Suicidality vs No Suicidality: No Controls, controlling for DASS					
-	Df	SumSq	MeanSq	F-value	P-value
Sui.Yes	1	0.43	0.43001	28.8241	0.00000014

Table S12. Two-way ANOVA comparing mean intrinsic network functional connectivity in suicidality vs no suicidality groups without healthy control participants and controlling for DASS

Intrinsic Connectivity Networks - ANOVA Suicidality vs No Suicidality: No Controls, controlling for DASS					
Response Var - ICNs	df	sumsq	meansq	statistic	P-value
Default_Sal_Mean	1	0.418804	0.418804	16.50417	0.000059205
Default_DAN_Mean	1	0.428073	0.428073	8.626469	0.003518779
Default_SomMot_Mean	1	0.370443	0.370443	13.54416	0.000267355
Default_Vis_Mean	1	0.150689	0.150689	4.930053	0.026993880
Limbic_SomMot_Mean	1	0.282699	0.282699	22.20286	0.000003468
Limbic_Vis_Mean	1	0.165208	0.165208	9.311778	0.002439866
Sal_Sal_Mean	1	0.574658	0.574658	11.9842	0.000598722
Sal_SomMot_Mean	1	0.666069	0.666069	25.14055	0.000000826
Sal_Vis_Mean	1	0.194543	0.194543	5.378188	0.020929680
DAN_Vis_Mean	1	0.61729	0.61729	13.25654	0.000309996
SomMot_Mean	1	0.477685	0.477685	19.51998	0.000013065
SomMot_Vis_Mean	1	0.363725	0.363725	19.65757	0.000012201
Vis_Vis_Mean	1	0.339601	0.339601	9.955564	0.001733873

Table S13. Two-way ANOVA comparing of suicidality vs no suicidality (with healthy control participants) controlling for DASS

Network Mean -_ANOVA Suicidality vs No Suicidality:with Controls, controlling for DASS					
	Df	SumSq	MeanSq	F-value	P-value
Suicidal	1	0.6393	0.63935	40.8948	0.0000000003

Table S14. Two-way ANOVA comparing of mean intrinsic network functional connectivity in suicidality vs no suicidality groups (with healthy control participants) controlling for DASS

Intrinsic Connectivity Networks - ANOVA Suicidality vs No Suicidality: with Controls, controlling for DASS					
Response Var - ICNs	df	sumsq	meansq	statistic	P-value
Default_Sal_Mean	1	0.546	0.546	21.258	0.000005009
Default_DAN_Mean	1	0.574	0.574	11.817	0.000631871
Default_SomMot_Mean	1	0.643	0.643	22.965	0.000002131
Default_Vis_Mean	1	0.336	0.336	10.830	0.001063629
Limbic_SomMot_Mean	1	0.441	0.441	30.682	0.000000047
Limbic_Vis_Mean	1	0.263	0.263	15.365	0.000099937
Sal_Sal_Mean	1	0.672	0.672	13.971	0.000205261
Sal_SomMot_Mean	1	0.723	0.723	27.421	0.000000234
Sal_Vis_Mean	1	0.374	0.374	10.295	0.001412255
DAN_Vis_Mean	1	0.964	0.964	20.876	0.000006067
SomMot_Mean	1	0.795	0.795	31.060	0.000000039
SomMot_Vis_Mean	1	0.586	0.586	29.871	0.000000070
Vis_Vis_Mean	1	0.471	0.471	13.889	0.000214207
