# Supplementary material for: The neural connectome of suicidality in adults with mood and anxiety disorders
Source: Nat Ment Health. 2024 Oct 4;2(11):1342–9. doi: 10.1038/s44220-024-00325-y (PMC11540851; doi:10.1038/s44220-024-00325-y)
Supplement: Supplementary file 2 — Reporting Summary [file 44220_2024_325_MOESM2_ESM.pdf]

nature portfolio

Corresponding author(s): ______________________

Last updated by author(s): ______________________

Reporting Summary

Nature Portfolio wishes to improve the reproducibility of the work that we publish. This form provides structure for consistency and transparency in reporting. For further information on Nature Portfolio policies, see our Editorial Policies and the Editorial Policy Checklist.

Please do not complete any field with "not applicable" or n/a. Refer to the help text for what text to use if an item is not relevant to your study.
For final submission: please carefully check your responses for accuracy; you will not be able to make changes later.

Statistics

For all statistical analyses, confirm that the following items are present in the figure legend, table legend, main text, or Methods section.

n/a	Confirmed	
☐	☒	The exact sample size (*n*) for each experimental group/condition, given as a discrete number and unit of measurement
☐	☒	A statement on whether measurements were taken from distinct samples or whether the same sample was measured repeatedly
☐	☒	The statistical test(s) used AND whether they are one- or two-sided<br>*Only common tests should be described solely by name; describe more complex techniques in the Methods section.*
☐	☒	A description of all covariates tested
☐	☒	A description of any assumptions or corrections, such as tests of normality and adjustment for multiple comparisons
☐	☒	A full description of the statistical parameters including central tendency (e.g. means) or other basic estimates (e.g. regression coefficient) AND variation (e.g. standard deviation) or associated estimates of uncertainty (e.g. confidence intervals)
☐	☒	For null hypothesis testing, the test statistic (e.g. *F*, *t*, *r*) with confidence intervals, effect sizes, degrees of freedom and *P* value noted<br>*Give P values as exact values whenever suitable.*
☒	☐	For Bayesian analysis, information on the choice of priors and Markov chain Monte Carlo settings
☒	☐	For hierarchical and complex designs, identification of the appropriate level for tests and full reporting of outcomes
☐	☒	Estimates of effect sizes (e.g. Cohen's *d*, Pearson's *r*), indicating how they were calculated

Our web collection on statistics for biologists contains articles on many of the points above.

Software and code

Policy information about availability of computer code

Data collection	
Data analysis	

For manuscripts utilizing custom algorithms or software that are central to the research but not yet described in published literature, software must be made available to editors and reviewers. We strongly encourage code deposition in a community repository (e.g. GitHub). See the Nature Portfolio guidelines for submitting code & software for further information.

Data

Policy information about availability of data

All manuscripts must include a data availability statement. This statement should provide the following information, where applicable:

- Accession codes, unique identifiers, or web links for publicly available datasets
- A description of any restrictions on data availability
- For clinical datasets or third party data, please ensure that the statement adheres to our policy

The data used in this study can be be available from the corresponding author upon reasonable request. Due to privacy and ethical requirements the data are not publicly available.

Research involving human participants, their data, or biological material

Policy information about studies with human participants or human data. See also policy information about sex, gender (identity/presentation), and sexual orientation and race, ethnicity and racism.

Reporting on sex and gender	We report that 309 (53.4%) participants were female.
Reporting on race, ethnicity, or other socially relevant groupings	Ethnicity data was not used as part of these analyses.
Population characteristics	Suicidal Participants: Mean age: 35.9 years; 148 (62.2%) female; 90 (37.8%) male. Non-Suicidal Participants: Mean age: 35.2 years; 161 (47.2%) female; 180 (52.8%) male.
Recruitment	Recruitment by public advertising which may lead to self-selection bias.
Ethics oversight	Ethics approval granted by Western Sydney Area Health Service Ethics Committee.

Note that full information on the approval of the study protocol must also be provided in the manuscript.

Field-specific reporting

Please select the one below that is the best fit for your research. If you are not sure, read the appropriate sections before making your selection.

☐ Life sciences ☒ Behavioural & social sciences ☐ Ecological, evolutionary & environmental sciences

For a reference copy of the document with all sections, see nature.com/documents/nr-reporting-summary-flat.pdf

Life sciences study design

All studies must disclose on these points even when the disclosure is negative.

Sample size	n/a
Data exclusions	n/a
Replication	n/a
Randomization	n/a
Blinding	n/a

Behavioural & social sciences study design

All studies must disclose on these points even when the disclosure is negative.

Study description	Data involves case-control design of quantitative data comprising neuroimaging results.
Research sample	Convenience sample of psychiatric and non-psychiatric individuals of mean age 35 years (54% female).
Sampling strategy	Sample is not necessarily representative. Recruited via advertising. No power analysis; largest study of its kind reported.
Data collection	Measures included Mini International Neuropsychiatric Interview , Depression Anxiety Stress Scale. Imaging acquired in a 3T GE Sigma Scanner. Those acquiring imaging data were blind to psychiatric status.
Timing	Data collecction occurred between Jan 2009 and Sept 2015.
Data exclusions	660 participants recruited, however of these 5 were missing relevant clinical data, 35 failed to complete the MRI, 39 excessive movement within the MRI, and 2 had artifacts or registrations issues in the scanner.
Non-participation	No recruited participants did not participate.
Randomization	No randomization because all participants underwent the same protocol

April 2023

Ecological, evolutionary & environmental sciences study design

All studies must disclose on these points even when the disclosure is negative.

Study description	n/a
Research sample	n/a
Sampling strategy	n/a
Data collection	n/a
Timing and spatial scale	n/a
Data exclusions	n/a
Reproducibility	n/a
Randomization	n/a
Blinding	n/a

Did the study involve field work? ☐ Yes ☒ No

Field work, collection and transport

Field conditions	n/a
Location	n/a
Access & import/export	n/a
Disturbance	n/a

Reporting for specific materials, systems and methods

We require information from authors about some types of materials, experimental systems and methods used in many studies. Here, indicate whether each material, system or method listed is relevant to your study. If you are not sure if a list item applies to your research, read the appropriate section before selecting a response.

Materials & experimental systems

n/a	Involved in the study	
☒	☐	Antibodies
☒	☐	Eukaryotic cell lines
☒	☐	Palaeontology and archaeology
☒	☐	Animals and other organisms
☒	☐	Clinical data
☒	☐	Dual use research of concern
☒	☐	Plants

Methods

n/a	Involved in the study	
☒	☐	ChIP-seq
☒	☐	Flow cytometry
☐	☒	MRI-based neuroimaging

Antibodies

Antibodies used	n/a
Validation	n/a

Eukaryotic cell lines

Policy information about cell lines and Sex and Gender in Research

Cell line source(s)	n/a
Authentication	n/a
Mycoplasma contamination	n/a
Commonly misidentified lines (See ICLAC register)	n/a

Palaeontology and Archaeology

Specimen provenance	n/a
Specimen deposition	n/a
Dating methods	n/a

☐ Tick this box to confirm that the raw and calibrated dates are available in the paper or in Supplementary Information.

Ethics oversight	n/a

Note that full information on the approval of the study protocol must also be provided in the manuscript.

Animals and other research organisms

Policy information about studies involving animals; ARRIVE guidelines recommended for reporting animal research, and Sex and Gender in Research

Laboratory animals	n/a
Wild animals	n/a
Reporting on sex	n/a
Field-collected samples	n/a
Ethics oversight	n/a

Note that full information on the approval of the study protocol must also be provided in the manuscript.

Clinical data

Policy information about clinical studies

All manuscripts should comply with the ICMJE guidelines for publication of clinical research and a completed CONSORT checklist must be included with all submissions.

Clinical trial registration	n/a
Study protocol	n/a
Data collection	n/a
Outcomes	n/a

Dual use research of concern

Policy information about dual use research of concern

Hazards

Could the accidental, deliberate or reckless misuse of agents or technologies generated in the work, or the application of information presented in the manuscript, pose a threat to:

No	Yes	
☒	☐	Public health
☒	☐	National security
☒	☐	Crops and/or livestock
☒	☐	Ecosystems
☒	☐	Any other significant area

Experiments of concern

Does the work involve any of these experiments of concern:

No	Yes	
☒	☐	Demonstrate how to render a vaccine ineffective
☒	☐	Confer resistance to therapeutically useful antibiotics or antiviral agents
☒	☐	Enhance the virulence of a pathogen or render a nonpathogen virulent
☒	☐	Increase transmissibility of a pathogen
☒	☐	Alter the host range of a pathogen
☒	☐	Enable evasion of diagnostic/detection modalities
☒	☐	Enable the weaponization of a biological agent or toxin
☒	☐	Any other potentially harmful combination of experiments and agents

Plants

Seed stocks	n/a
Novel plant genotypes	n/a
Authentication	n/a

ChIP-seq

Data deposition

☐ Confirm that both raw and final processed data have been deposited in a public database such as GEO.

☐ Confirm that you have deposited or provided access to graph files (e.g. BED files) for the called peaks.

Data access links<br>*May remain private before publication.*	n/a
Files in database submission	n/a
Genome browser session<br>(e.g. UCSC)	n/a

Methodology

Replicates	n/a
Sequencing depth	n/a
Antibodies	n/a
Peak calling parameters	n/a
Data quality	n/a

Software: n/a

Flow Cytometry

Plots

Confirm that:

- [ ] The axis labels state the marker and fluorochrome used (e.g. CD4-FITC).
- [ ] The axis scales are clearly visible. Include numbers along axes only for bottom left plot of group (a 'group' is an analysis of identical markers).
- [ ] All plots are contour plots with outliers or pseudocolor plots.
- [ ] A numerical value for number of cells or percentage (with statistics) is provided.

Methodology

Sample preparation: n/a

Instrument: n/a

Software: n/a

Cell population abundance: n/a

Gating strategy: n/a

- [ ] Tick this box to confirm that a figure exemplifying the gating strategy is provided in the Supplementary Information.

Magnetic resonance imaging

Experimental design

Design type: Case control design

Design specifications: Comparison of suicidal and non-suicidal adults

Behavioral performance measures: Instrinsic resting state images. No other behavioral measures acquired.

Imaging type(s): GE Sigma Twinspeed-HDXT MR Scanner

Field strength: 3T

Sequence & imaging parameters: Echo planner imaging sequence conducted with the following parameters: repetition time = 2,500ms, echo time = 27 = Sms, matrix = 64x64, FOV = 24cm, flip angle = 90 degrees, 120 volumes.

Area of acquisition: Whole brain.

Diffusion MRI: [ ] Used [x] Not used

Preprocessing

Preprocessing software: SPM12 running on MATLAB with some use of FSL modalities

Normalization: Normalisation was done using FSL's FMRIB nonlinear registration tool

Normalization template: Montreal Neurological Institute (MNI) 152 T1 2x2x2mm3

Noise and artifact removal: The Volterra expansion of twenty-four realignment parameters were modelled for each fMRI task as well as white matter and cerebrospinal fluid signals were derived and regressed from the first level GLM models. Also, outlier volumes were identified based on framewise displacement—movement of the head from one volume to the next—of 0.3mm or greater or as a difference in scaled signal intensity greater than 10. Scrubbing was then performed by creating temporal masks for the two volumes before and one after the movement outlier and using these as regressors of no interest in the first level statistical models

Volume censoring: Volumes with a fractional displacement (FD) of 0.3mm or higher or with a signal scale intensity difference of more than 10 between volumes were excluded as movement outliers. The volumes either side of the outlier were also excluded.

Statistical modeling & inference

Model type and settings: Functional connectomes generated for each participant were analyzed to test differences in whole-brain intrinsic connectivity between suicidal and non-suicidal participants using the Network Based Statistic software.

Effect(s) tested: Connectivity Group difference across the connectome in both directions (Suicidal > Non-suicidal and Non-suicidal > Suicidal). Clinical diagnosis (individual diagnostic categories), age in years, gender and years of education were also included in the design matrix as covariates of no interest

Specify type of analysis: ☒ Whole brain ☐ ROI-based ☐ Both

Statistic type for inference (See [Eklund et al. 2016](https://www.pnas.org/doi/10.1073/pnas.1602413113))

The NBS is a validated, non-parametric method that accounts for multiple comparisons by basing hypothesis tests on interconnected subnetworks rather than on individual connections. In this approach, t-tests are used to discover links between parcels that differ between groups. Connections that pass the t-statistic threshold are combined to form networks. Each network is tested for significance by comparing it to networks formed through random permutation.

Correction

Permutation testing conducted with family-wise corrected p-value.

Models & analysis

n/a	Involved in the study
☐	☒ Functional and/or effective connectivity
☒	☐ Graph analysis
☒	☐ Multivariate modeling or predictive analysis

Functional and/or effective connectivity

Intrinsic functional connectivity data was extracted by concatenating the residual time series of the functional scans after modeling out the task related effects and noise and motion effects. Using the general linear model framework separately for each task, the blood oxygen-level dependent (BOLD) responses was modelled for each experimental condition within each fMRI task. Intrinsic signal images were derived by modeling and regressing out the variance in BOLD signal associated with each of the stimuli in the tasks as a covariate. Each participant’s average time series was extracted from 400 cortical regions and 36 subcortical regions which were clustered into 7 intrinsic connectivity networks (ICNs). Then, the BOLD time series from the intrinsic connectivity data was extracted for each parcel and correlated pair-wise with all other parcels. Finally, a Fisher-Z transform was applied to produce a 436x436 functional correlation matrix (i.e. functional connectome) for every participant. ICNs were defined as per the Schaefer parcellation or as belonging to subcortical network. The labelled networks were the default mode network, dorsal attention network, ventral attention network, cognitive control network, visual network, somatomotor network, limbic network, or subcortex.

Graph analysis

n/a

Multivariate modeling and predictive analysis

n/a

This checklist template is licensed under a Creative Commons Attribution 4.0 International License, which permits use, sharing, adaptation, distribution and reproduction in any medium or format, as long as you give appropriate credit to the original author(s) and the source, provide a link to the Creative Commons license, and indicate if changes were made. The images or other third party material in this article are included in the article's Creative Commons license, unless indicated otherwise in a credit line to the material. If material is not included in the article's Creative Commons license and your intended use is not permitted by statutory regulation or exceeds the permitted use, you will need to obtain permission directly from the copyright holder. To view a copy of this license, visit http://creativecommons.org/licenses/by/4.0/
